# Mutation analysis of the WNT4 gene in Han Chinese women with premature ovarian failure

**DOI:** 10.1186/1477-7827-9-75

**Published:** 2011-05-30

**Authors:** Beili Chen, Peisu Suo, Binbin Wang, Jing Wang, Lu Yang, Sirui Zhou, Ying Zhu, Xu Ma, Yunxia Cao

**Affiliations:** 1 Reproductive Medicine Center, First Affiliated Hospital, Anhui Medical University, Hefei, PR China; 2 National Research Institute for Family Planning, Beijing, 100081, PR China; 3 Graduate School, Peking Union Medical College, Beijing, PR China; 4 World Health Organization Collaborating Centre for Research in Human Reproduction, Beijing, PR China

## Abstract

**Background:**

The *WNT4 *gene plays an important role in female sex determination and differentiation. It also contributes to maintaining of the ovaries and the survival of follicles.

**Methods:**

We sequenced the coding region and splice sites of *WNT4 *in 145 Han Chinese women with premature ovarian failure (POF) and 200 healthy controls.

**Results:**

Only one novel variation, in Exon 2 (195C > T), was detected among the women with POF. However, this synonymous variation did not result in a change in amino acid sequence (65 Asp > Asp). No further variants were found in any of the samples.

**Conclusion:**

Although we cannot provide any evidence that it is a possible disease-causing gene, this study is the first attempt to investigate the possible role of *WNT4 *in Han Chinese women with POF.

## Background

Premature ovarian failure (POF) is a rare disease that severely affects the reproductive health and endocrine balance of 1% of all women [1]. It is characterized by abnormal follicular genesis, development or apoptosis. POF presents clinically as primary amenorrhea in women with abnormal prepubertal onset, or as spontaneous secondary amenorrhea in women with premature depletion of ovarian follicles/arrested folliculogenisis. The etiological factors and pathogenesis of POF are extremely heterogeneous. As a severe complication in patients after treatments for ovarian neoplasms or autoimmune diseases, the damage caused by iatrogenic factors (surgery, chemotherapy, radiations) accounts for many cases. However, the causes are still unclear in most cases of POF (especially idiopathic form). Genetic factors are known to provide a significant etiological component in recent studies. Apart from studying X chromosome deletions and X/autosome translocations in patients, research has focused increasingly on some candidate genes involved in ovarian failure in animal models, such as *Fshr *and *Foxl2 *[2].

*Wnt4*, which encodes a secreted extracellular signaling protein, is known to be involved in multiple developmental processes, such as the formation of the kidney, adrenal gland and gonads [3-5]. Many studies have demonstrated that this gene plays an important role in mammalian female sexual differentiation. It is downregulated in the testis and upregulated in the ovary at 11.5 days post-coitum in the mouse by activating Frizzled-4, expressed on luteal cell membranes to activate distinct signaling cascades [5]. The inactivation of *Wnt4 *led to ovarian dysgenesis with early production of testosterone by mesenchymal cells and formation of male internal genitalia, including testicular tubules and spermatogonia in XX mice [6,7].

Recently, attention has been paid to the contribution of *WNT4 *for the maintenance of ovaries and survival of follicles in the mouse and humans. *WNT4 *is expressed in the bipotential embryonic gonad of both sexes, and increases when small primary follicles are formed during mid-pregnancy. Vainio *et al. *observed that the number of oocytes in *Wnt4 *mutant mouse ovaries was abnormally low at birth [5]. Although *Wnt4 *has not been shown to affect the number of primordial germ cells in the undifferentiated gonad, it is thought to act as an oocyte survival factor during female embryogenesis [8]. Compared with wild-type mice, *Wnt4 *deficient mice had a markedly enhanced rate of oocyte apoptosis [9,10]. There are two oocyte-specific transcriptional factors, FIGLA and NOBOX, acting downstream of *WNT4 *signaling, which are associated with POF [11,12]. Therefore, we carried out mutational screening of the *WNT4 *gene in Han Chinese women with POF.

## Methods

### Patient information

One hundred and forty-five unrelated women with idiopathic POF and 200 healthy controls were recruited from the Reproductive Medical Center of the First Affiliated Hospital, Anhui Medical University, P. R. China. All the participants were the Han people (China's main nationality) in three generations without minority and signed the informed consent. The diagnostic criteria for POF were defined as follows. (1) Women showed primary amenorrhea or spontaneous secondary amenorrhea for more than 6 months before the age of 40 years. (2) Repeated elevated levels of follicle stimulating hormone (FSH; >30 mIU/mL) and low plasma estradiol levels. (3) Normal karyotype 46, XX. (4) No history of pelvic surgery, chemotherapy or autoimmune diseases. All the healthy controls had regular menstrual cycles, normal FSH levels and denied any history of a medical disease related to ovarian function.

The mean age of patients with POF at the time of study, menarche and amenorrhea were 30.4 ± 6.51 years, 14.5 ± 1.98 years and 25.9 ± 6.90 years, respectively. The mean age of the controls was 31.2 ± 4.50 years at screening (range 21-52). All subjects had a normal growth history and weight based on a physical examination. Patients with POF had a mean serum FSH level of 74.92 ± 30.52 mIU/mL (LH 32.78 ± 15.75 mIU/mL) and presented with primary amenorrhea (17, 11.7%) or secondary amenorrhea (128, 88.3%), accompanied by some endocrine disorders such as hectic fever, night sweating, anxiety, fatigue or vaginal dryness. No patient was found with hyperandrogenism. Ultrasonography showed that most of the ovaries in women with POF were smaller than normal with few growing follicles or even no follicles. In women with primary amenorrhea, gonadal dysgenesis was documented by the finding of streak ovaries. The possibilities of other causes of primary amenorrhea, such as congenital absence of the vagina, androgen insensitivity syndrome, and Turner's syndrome, had already been ruled out through gynecological examinations, transvaginal ultrasound checks, and endocrine and chromosomal studies.

### DNA analysis

Genomic DNA was extracted from 200 μl samples of peripheral blood leukocytes using TIANamp Blood DNA kits (TIANGEN, Beijing, P.R. China). The entire coding region and splice sites of the *WNT4 *gene (comprising 5 exons) were divided into four parts to be amplified by polymerase chain reaction (PCR) using primers designed by Primer 5.0 software (http://www.premierbiosoft.com/primerdesign/index.html). The sequences of primers and specific conditions for PCR amplification are available on request. All the PCR products were directly sequenced using an ABI 3730XL automated sequencer (Applied Biosystems, Foster City, CA, USA) according to the manufacturer's protocol.

## Results

PCR was performed to amplify the coding region of *WNT4 *and the sequencing data was collected and analyzed for all cases and controls. Only one novel variation was detected among the 145 patients with POF. This synonymous variation in Exon 2 (195C > T), which was not identified in the control population, did not result in a change in amino acid sequence (65 Asp > Asp). No further variants were found in any of the samples.

## Discussion

Apart from the accepted view that *WNT4 *is a key regulator of gonadal determination and differentiation, its mRNA and protein are present in human ovaries during fetal development and at different stages of follicular development in adult ovaries [10]. Some functional analyses revealed that deficiencies in the *WNT4 *gene might affect the development of ovaries and follicles. Vainio *et al. *observed that the ovaries of *Wnt4 *mutant female mice had only a few degenerating oocytes [5]. Yao *et al. *found that germ cells in the ovarian cortex were almost completely absent in both *Wnt4 *and *Fst *null gonads and concluded that *Wnt4 *might play a critical role in maintaining germ cell survival in the ovary [9]. Similarly, Jääskeläinen *et al. *also revealed that *Wnt4*-deficient mice had a markedly enhanced rate of oocyte apoptosis [10].

Based on the above-mentioned studies and views, researchers began to study this gene from different aspects in human diseases, mainly involving Müllerian Duct abnormalities. A French collaborative study identified a new L12P mutation with Exon 1 of the *WNT4 *gene from a patient who presented with uterine hypoplasia and follicle depletion [13]. Biason-Lauber *et al. *also found that one girl with absence of a uterus, androgen excess and lack of follicles in her left ovary carried a novel loss-of-function dominant negative mutation in *WNT4 *[14]. Moreover, a homozygous missense mutation in the human *WNT4 *gene was identified in a novel autosomal-recessive syndrome SERKAL which consists of female to male sex reversal and renal, adrenal, and lung dysgenesis and is associated with additional developmental defects [15]. Therefore, we tried to determine whether Han Chinese women with POF might show a genetic background linked to the *WNT4 *gene. However, we did not observe any causal variant: only a silent mutation. *Wnt *genes have been highly conserved throughout evolution and play pivotal roles in a wide variety of physiological events during embryogenesis. We supposed that this conservatism and importance might determine the stability of the *WNT4 *gene. In addition, Jääskeläinen also pointed towards species-specific differences in the expression patterns of *Wnt4 *when comparing the results from mice and humans [10].

In conclusion, this study was the first attempt to investigate the possible role of *WNT4 *gene in Han Chinese women with POF, although we could not provide any evidence that it caused the disease. Therefore, more samples from different populations should be analyzed.

## Competing interests

The authors declare that they have no competing interests.

## Authors' contributions

BC, YL and YZ collected all samples and performed the clinical tests. PS and JW carried out the molecular genetic studies. BC and SZ drafted the manuscript. BW, XM and YC participated in its design and coordination and helped to draft the manuscript. All authors read and approved the final manuscript.
